# What is biology research for?

**DOI:** 10.1371/journal.pbio.3004075

**Published:** 2026-10-15

**Authors:** Ran Blekhman

**Affiliations:** Section of Genetic Medicine, Department of Medicine, University of Chicago, Chicago, Illinois, United States of America

## Abstract

In the age of generative artificial intelligence (AI), the assumption that writing a paper requires doing science no longer holds true. Before deciding how to respond to the surge of AI-generated papers, the field needs to state what biology research is actually for.

The number of scientific publications has been growing steadily for decades, but starting in late 2022, the volume of manuscripts submitted to academic journals escalated with unprecedented speed. Monthly submissions to arXiv have nearly doubled [1], and the timing of this inflection matches the public rollout of the first widely used generative artificial intelligence (AI) systems. Although some of this surge represents a trend that began before AI, the acceleration of the past four years is hard to explain without considering automated text generation [2]. ArXiv has been actively trying to address the issue: in 2025 it stopped accepting review and position papers in computer science that had not already passed peer review [3], and in 2026 it announced a 1-year submission ban for authors whose papers contain unchecked AI content such as hallucinated references or leftover chatbot instructions [4]. These responses are reasonable, but they address symptoms rather than the underlying problem.

Put bluntly, it is now straightforward to generate a plausible scientific paper without doing any actual research. Scientists have to come to terms with what this means: the paper, the artifact of research that the biology research community has used to assess, fund, hire, and promote for a century, has come loose from the science it was meant to represent.

I have spent a long time thinking about what this means for biology without much progress, until, as is often the case, I gained clarity by reading the thoughts of someone much smarter than me. In a recent essay, Terence Tao wrote about mathematics in the age of AI, and argued that a field grappling with AI-generated results first has to say plainly what the goals of the field’s research actually are [5].

I believe the same exercise is due in biology. What is biology research for? This is of course a complicated exercise, and not one any single scientist can do on behalf of the field. Nevertheless, here is my own first pass at it, offered as a starting point for a conversation. First, we do biology research to explain how living systems work at every scale from molecules to ecosystems. Second, we do biology research to improve human health, whether directly through therapy or via the longer chain that runs from a basic finding to a medicine decades later. Third, we do biology research to build the resources that facilitate the field, from datasets and model organisms to reference databases, methods, and software. Fourth, we do biology research to train the next generation of scientists who will do this work when we no longer can. And fifth, we do biology research to keep the trust of a public that pays for almost all of it (this goal is the least frequently named, but arguably the most important one).

For most of the 20th century, producing a scientific paper advanced at least some of these goals. For example, a paper describing a mechanistic finding also generated a dataset, trained a student in its methods, and potentially laid the groundwork for eventual therapeutic applications. Because manuscripts reflected real progress towards our field’s goals, our assessment systems began relying on them as primary metrics: review panels, search committees and promotion decisions gradually led to scientific quality being collapsed into manuscript counts, venue metrics and citations. This convergence was not defensible on first principles, and by the late 2000s it had been challenged repeatedly [6]. I am not defending this proxy, which is obviously flawed; still, this style of evaluating science took hold because a paper took effort to produce, and that effort served as evidence that at least one goal on the list had been advanced.

Generative AI has broken this already-flawed proxy completely. A plausible paper can now be produced in a day, and the process of producing it does not necessarily enhance knowledge, build a resource, train a student, or advance any other goal of biology research. This is a crisis, and it is one the field has not yet begun to grapple with.

Admittedly, scientific manuscript quality has long been uneven; paper mills, fabricated data, and low-quality publications have been a consistent feature of the scientific record since well before the arrival of generative AI. Thus, one could argue that machine-written manuscripts do not present a novel challenge. However, I believe there is a clear distinction: producing a paper, even a neglected one, still requires some labor and resources, therefore indicating some level of genuine scientific engagement; even a marginal paper likely contributes to some of biology’s goals. Automated text generation eliminates this link between manuscript production and authentic academic endeavor.

Another objection is that the surge in AI-generated manuscripts in biology is smaller than in fields such as machine learning or mathematics, so perhaps it is not really a crisis. This is partly true, because AI has not yet substantially increased productivity in biology, where the bottlenecks are structural and experimental rather than computational [7]. The surge is nevertheless just beginning. Computational biology already sees it, because AI can fabricate a computational paper end to end. And, in my opinion, the surge will just get worse. This is because, in biology research, there are no brakes on the system: scientists will keep generating more and more papers because their careers depend on it; publishers will keep publishing them and collecting fees to increase their revenue; and AI companies will happily keep selling the tokens that enable it.

Therefore, we have to be deliberate about establishing a new way to evaluate biology research. The response cannot be that we stop evaluating ourselves. In the absence of a deliberate response, my prediction is that evaluation will fall back on older, cruder proxies, such as journal prestige and impact factor, which would undo the past decade of research assessment reform. The San Francisco Declaration on Research Assessment and the Leiden Manifesto [8] pushed the field to move away from journal name and citation counts as measures of quality, and made real, if slow, progress. AI-driven uncertainty about what a paper represents will reverse that progress. Instead, we could use this surge to acknowledge that paper counts are meaningless, and, as a field, abandon the current incentive system and return our focus to the substance of research.

Goodhart’s law is often stated as the observation that, when a measure becomes a target, it stops being a good measure [9,10]. ‘Publish more papers’ has been a target in biology for decades. The biology research community needs to define new ways to evaluate science that are aligned with its goals. This is work for the entire field: journals, funders, universities, and institutions. We, as a field, have got to find a better goal. Maybe, as a starting point, we should start by asking ourselves: what is biology research for?
